# Interaction between peroxisome proliferator-activated receptor gamma polymorphism and obesity on type 2 diabetes in a Chinese Han population

**DOI:** 10.1186/s13098-017-0205-5

**Published:** 2017-01-19

**Authors:** Xiaohui Lv, Li Zhang, Jiayu Sun, Zhigang Cai, Qing Gu, Ruipeng Zhang, Aiyun Shan

**Affiliations:** 1 Shenzhen Futian District Traditional Chinese Medicine Hospital, No. 6001 in North Central Avenue, Futian District, Shenzhen, 518033 Guangdong China; 2 Key Lab for New Drugs Research of TCM, Research Institute of Tsinghua University in Shenzhen, Shenzhen, Guangdong China

**Keywords:** Type 2 diabetes mellitus, PPAR, Polymorphism, Obesity, Interaction

## Abstract

**Aims:**

The aim of this study was to investigate the association of four single nucleotide polymorphisms (SNPs) of peroxisome proliferator-activated receptor gamma (PPARG) with type 2 diabetes mellitus (T2DM) risk and additional role of gene-obesity interaction.

**Methods:**

Four SNPs were selected for genotyping in the case–control study: rs1805192, rs709158, rs3856806 and rs4684847. Generalized multifactor dimensionality reduction (GMDR) model and logistic regression was used to examine the interaction between SNP and obesity on T2DM, odds ratio (OR) and 95% confident interval (95% CI) were calculated.

**Results:**

T2DM risk was significantly higher in individuals with rs1805192-G allele (*p* < 0.05). The carriers of G allele of the rs1805192 polymorphism revealed increased T2DM risk than those with CC variants (CG + GG versus CC, adjusted OR (95% CI) 1.76 (1.45–2.06), p < 0.001). T2DM risk was also significantly higher in individuals with rs3856806-T allele (*p* < 0.05). The carriers of T allele of the rs3856806 polymorphism revealed increased T2DM risk than those with CC variants (CT + TT versus CC, adjusted OR (95% CI) 1.25 (1.17–1.76), *p* < 0.001). There was a significant two-locus model (p = 0.0107) involving rs1805192 and obesity. Obese subjects with CG or GG genotype have the highest T2DM risk, compared to subjects with CC genotype and normal BMI (OR 2.40, 95% CI 1.68–3.63).

**Conclusions:**

Our results support an important association between rs1805192 and rs3856806 minor allele (G allele) of PPARG and increased T2DM risk, the interaction analysis shown a combined effect of G- obesity interaction between rs1805192 and obesity on increased T2DM risk.

## Background

Diabetes mellitus (DM) is a group of common metabolic disorders that share the phenotype of hyperglycemia. Globally, about 5.4% of adult population has been estimated to have type 2 diabetes mellitus (T2DM) [1]. Several types of diabetes mellitus exist and are caused by a complex interaction of genetics and environmental factors [2]. Type 2 diabetes has a strong genetic component. There is evidence that the Pro12Ala polymorphism is linked to obesity and T2DM [3].

The peroxisome proliferator-activated receptor gamma (PPARG) is now recognized to play a main PPARG entered the spotlight as a major player in metabolic regulation in the early 1990s [4] through the discovery of thiazolidinediones (TZDs) as potent synthetic insulin-sensitizing drugs. TZDs quickly passed clinical trials and became front-line agents in the treatment of type II diabetes for their robust insulin-sensitizing and glucose-lowering actions, although their popularity has recently decreased in response to safety concerns. Activation of PPARG results in systemic insulin sensitization through complex mechanisms involving multiple. PPARG was the first gene reproducibly associated with T2DM. The association between the substitution of alanine by proline at codon 12 of PPARG2 (Ala12 allele) and the risk for T2DM has been widely studied since Yen et al. [5], first reported this polymorphism. Some studies indicated that rs1805192 SNP plays a key role in regulating the expression of numerous genes involved in lipid metabolism, metabolic syndrome, inflammation, and atherosclerosis [6, 7]. Some environmental risk factors for T2DM were suggested, such as obesity, which was reported in different populations [8–10]. However, till now, no study focused on the impact of gene- environment interaction between PPARG and obesity on T2DM risk in Chinese population. So the aim of this study was to investigate the association of PPARG, and additional PPARG gene- obesity interaction with T2DM risk based on a Chinese population.

## Methods

### Subjects

This was a case–control study. Chinese patients with T2DM were consecutively recruited between January 2012 and December 2013 from the Shenzhen Futian District traditional Chinese medicine hospital. A total of 1297 subjects consist of 647 T2DM patients and 650 normal controls were included in this study, including 606 males and 691 females. The mean age of all subjects was 54.3 ± 15.8 years old. The selected subjects were similar to those who were not selected in terms of age, sex, smoking status and alcohol consumption. Informed consent was obtained from all participants.

### Body measurements

Data on general demographic information and lifestyle information for all participants were obtained using a standard questionnaire administered by trained staffs. Body weight, height and waist circumference (WC) was measured, and body mass index (BMI) was calculated as weight in kilograms divided by the square of the height in meters. Cigarette smokers were those who self-reported smoking cigarettes at least once a day for 1 year or more [11]. Alcohol consumption was expressed as the sum of milliliters of alcohol per week from wine, beer, and spirits [12]. Blood samples were collected in the morning after at least 8 h of fasting. All plasma and serum samples were frozen at −80 °C until laboratory testing. Plasma glucose was measured using an oxidase enzymatic method. Concentrations of high-density lipoprotein (HDL)-cholesterol and triglyceride (TG) were assessed enzymatically by an automatic biochemistry analyzer (Hitachi Inc, Tokyo, Japan) using commercial reagents. All analysis was performed by the same lab.

### Genomic DNA extraction and genotyping

We selected SNPs within PPARG according to the following methods: (1) more studied SNPs, such as rs1805192, which was more studied in previous studies; (2) we also selected the others SNP of PPARG, in order to find new SNP associated with T2DM in Chinese population. Four SNPs were selected for genotyping in this study: rs1805192, rs709158, rs3856806, rs4684847. Four SNPs were detected by Taqman fluorescence probe, and probe sequences of four SNPs were shown in Table 1. Genomic DNA from participants was extracted from EDTA-treated whole blood, using the DNA Blood Mini Kit (Qiagen, Hilden, Germany) according to the manufacturer’s instructions. ABI Prism7000 software and allelic discrimination procedure was used for genotyping of fore-mentioned four SNPs. A 25 μl reaction mixture including 1.25 μl SNP Genotyping Assays (20×), 12.5 μl Genotyping Master Mix (2×), 20 ng DNA, and the conditions were as follows: initial denaturation for 10 min and 95 °C, denaturation for 15 s and 92 °C, annealing and extension for 90 s and 60 °C, 50 cycles.

**Table 1:** Probe sequence for four SNPs used for Taqman fluorescence probe analysis

SNP	Rs number	Chromosome	Functional consequence	Position	Probe sequence
C1341T	rs3856806	3	Intron variant	12,415,557	5′-GGTTGACACAGAGATGCCATTCTGG[C/G] CCACCAACTTTGGGATCAGCTCCGT-3′
Intron A>G	rs709158	3	Intron variant	12,403,176	5′-AGATACGGGGGAGGAAATTCACTGG[A/G] TTTTACAATATATTTTTCAAGGCAA-3′
Pro12Ala	rs1805192	3	Missense	12,361,238	5′-ACCTCAGACAGATTGTCACGGAACA[C/T] GTGCAGCTACTGCAGGTGATCAAGA-3′
Intron C>T	rs4684847	3	Intron variant	12,326,337	5′-ATTTATTTAAATCATCTCTAATTCT[C/T] ACAACTCCGAAAAGATAAGAAAACA-3′

### Diagnostic criteria

Diagnosis of diabetes at baseline for a fasting glucose was ≥126 mg/dl (7.0 mmol/l) or if hypoglycemic therapy (oral agents or insulin) had been executed. During the follow-up, the criteria for the diagnosis of T2DM included a fasting glucose ≥126 mg/dl (7.0 mmol/l), or a 2 h postprandial blood glucose ≥200 mg/dl (11.0 mmol/l), or if hypoglycemic therapy (oral agents or insulin) had been started in the interim [13].

Obesity was defined by using WHO criteria for Asian populations: BMI value ≥ 28 kg/m^2^ [14].

### Statistical analysis

The means and SD (standard deviation) was calculated for normally distributed continuous variables, and compared using t test. Percentages were calculated for categorical variable, and compared between case and control group participants using a Chi square test. Genotype and allele frequencies were obtained by direct count. Genotype distributions in T2DM patients and controls were evaluated by χ^2^ test using SPSS (version 19.0; SPSS Inc., Chicago, IL). Hardy–Weinberg equilibrium (HWE) was performed by using SNPStats (available online at http://bioinfo.iconcologia.net/SNPstats). Logistic regression model was used to examine the interaction between SNP and obesity on T2DM in additive model, odds ratio (OR) and 95% confident interval (95% CI) were calculated. Odds were adjusted for gender, age, smoke and alcohol status, high fat diet, low fiber diet, TC, TG and HDL. Generalized multifactor dimensionality reduction (GMDR) [15] was used to investigate the gene- environment interaction, cross-validation consistency, the testing balanced accuracy, and the sign test, to assess each selected interaction were calculated. The cross-validation consistency score is a measure of the degree of consistency with which the selected interaction is identified as the best model among all possibilities considered. Testing-balanced accuracy is a measure of the degree to which the interaction accurately predicts case–control status, and yields a score between 0.50 (indicating that the model predicts no better than chance) and 1.00 (indicating perfect prediction). Finally, the sign test, or permutation test (providing empirical p values), for prediction accuracy can be used to measure the significance of an identified model.

## Results

A total of 1297 subjects including 647 T2DM patients and 650 normal controls were included in this study, including 606 males and 691 females. The mean age of all subjects was 54.3 ± 15.8 years old. Participants characteristics stratified by T2DM cases and controls are shown in Table 2. The distributions of males, smoking, alcohol consumption, high fat diet, low fiber diet and mean of age were not different between cases and controls. The means of BMI, WC, TG, TC and HDL were significantly different between cases and controls.

**Table 2:** General characteristics of study participants in T2DM case and control group

Variables	T2DM cases group (n = 647)	Control group (n = 650)	*p* values
Age (years)	56.7 ± 14.8	55.4 ± 14.2	0.107
Males N (%)	310 (47.9)	296 (45.5)	0.391
Smoke N (%)	153 (23.6)	174 (26.8)	0.257
Alcohol consumption N (%)	144 (22.2)	136 (20.9)	0.559
High fat diet N (%)	106 (16.4)	114 (17.5)	0.579
Low fiber diet N (%)	118 (18.2)	96 (14.8)	0.092
WC (cm)	89.1 ± 15.8	86.8 ± 16.5	0.010
BMI (kg/m^2^)	25.8 ± 6.8	23.3 ± 6.6	<0.001
TG (mmol/l)	2.1 ± 0.67	1.8 ± 0.70	<0.001
TC (mmol/l)	5.3 ± 1.2	4.7 ± 1.1	<0.001
HDL (mmol/l)	1.21 ± 0.33	1.32 ± 0.27	<0.001

Median and inter quartile for TG; mean ± standard deviation for age, FPG, TC, HDL-C

*TC* total cholesterol, *HDL* high density lipoprotein, *FPG* fast plasma glucose, *TG* triglyceride

All genotypes were distributed according to Hardy–Weinberg equilibrium (all p values were more than 0.05). There were significant differences in rs1805192 and rs3856806 alleles and genotypes distributions between cases and controls (Table 3). The frequency for G allele of rs1805192 was higher in cases (49.0% in T2DM patients and 37.5% in controls, p < 0.001). Logistic analysis showed that T2DM risk was significantly higher in individuals with rs1805192-G allele (p < 0.05). The carriers of G allele of the rs1805192 polymorphism revealed increased T2DM risk than those with CC variants (CG + GG versus CC, adjusted OR (95% CI) 1.76 (1.45–2.06), p < 0.001). The frequency for T allele of rs3856806 was higher in cases (50.2% in T2DM patients and 38.9% in controls, p < 0.001). Logistic analysis showed that T2DM risk was significantly higher in individuals with rs3856806-T allele (p < 0.05). The carriers of T allele of the rs3856806 polymorphism revealed increased T2DM risk than those with CC variants (CT + TT versus CC, adjusted OR (95% CI) 1.25 (1.17–1.76), p < 0.001). However, we did not find any significant association between other two SNPs and T2DM risk before or after covariates adjustment.

**Table 3:** Genotype and allele frequencies of four SNPs between T2DM case and control group

SNPs	Genotypes and Alleles	Frequencies N(%)		OR (95% CI)^a^	*p* values	HWE test
		Case (n = 647)	Control (n = 650)			
rs3856806	CC	322 (49.8)	397 (61.1)	1.00	<0.001	0.379
	CT	256 (39.6)	217 (33.4)	1.12 (1.06–1.58)		
	TT	69 (10.7)	36 (5.5)	1.93 (1.34–2.71)		
	CT + TT	325 (50.2)	253 (38.9)	1.25 (1.17–1.76)	<0.001	
	C	900 (69.6)	1011 (77.8)		<0.001	
	T	394 (30.4)	289 (22.2)			
rs709158	AA	341 (52.7)	373 (57.4)	1.00	0.128	0.165
	AG	243 (37.6)	230 (35.4)	1.06 (0.95–1.37)		
	GG	63 (9.7)	47 (7.2)	1.10 (0.86–1.61)		
	AG + GG	306 (47.3)	277 (42.6)	1.07 (0.92–1.43)	0.090	
	A	925 (71.5)	976 (75.1)		0.039	
	G	369 (28.5)	324 (24.9)			
rs1805192	CC	330 (51.0)	406 (62.5)	1.00	<0.001	0.385
	CG	262 (40.5)	220 (33.8)	1.42 (1.61–1.84)		
	GG	55 (8.5)	24 (3.7)	2.13 (1.72–2.93)		
	CG + GG	317 (49.0)	244 (37.5)	1.76 (1.45–2.06)	<0.001	
	C	922 (71.2)	1032 (79.4)		<0.001	
	G	372 (28.8)	268 (20.6)			
rs4684847	CC	346 (53.5)	379 (58.3)	1.00	0.203	0.277
	CT	250 (38.6)	228 (35.1)	1.08 (0.91–1.36)		
	TT	51 (7.9)	43 (6.6)	1.04 (0.82–1.53)		
	CT + TT	301 (46.5)	271 (41.7)	1.07 (0.89–1.39)	0.080	
	C	942 (72.8)	986 (75.8)		0.076	
	T	352 (27.2)	314 (24.2)			

^a^Adjusted for gender, age, smoke and alcohol consumption status, high fat diet, low fiber diet, TC and HDL

We employed the GMDR analysis to assess the impact of the PPARG gene- obesity interaction on T2DM risk, after adjustment for gender, age, smoke and alcohol consumption status, high fat diet, low fiber diet, TC and HDL. Table 4 summarizes the results obtained from GMDR analysis for two- to five-locus models. There was a significant two-locus model (p = 0.0107) involving rs1805192 and obesity, indicating a potential gene–environment interaction among rs1805192 and obesity. Overall, the two- locus models had a cross-validation consistency of 10 of 10, and had the testing accuracy of 62.17%.

**Table 4:** Best gene- obesity interaction models, as identified by GMDR

Locus no.	Best combination	Cross-validation consistency	Testing accuracy	p values*
2	rs1805192 Obesity	10/10	0.6217	0.0107
3	rs1805192 rs3856806 Obesity	9/10	0.5577	0.1719
4	rs1805192 rs3856806 rs709158 Obesity	8/10	0.5590	0.0547
5	rs1805192 rs3856806 rs709158 rs4684847 Obesity	7/10	0.4958	0.3770

* Adjusted for gender, age, smoke and alcohol consumption status, high fat diet, low fiber diet, TC and HDL

In order to obtain the odds ratios and 95% CI for the joint effects of rs1805192 genotype and obesity on T2DM, we conducted interaction analysis between rs1805192 and obesity. We found that obese subjects with CG or GG genotype have the highest T2DM risk, compared to subjects with CC genotype and normal BMI (OR 2.40, 95% CI 1.68–3.63), after adjustment for gender, age, smoke and alcohol status, high fat diet, low fiber diet, TC and HDL (Table 5).

**Table 5:** Interaction analysis for rs1805192 and obesity on T2DM by using logistic regression

Rs1805192	Obesity	OR (95% CI)^a^	*p* values
CC	No	1.00	–
CG or GG	No	1.21 (1.08–1.47)	0.020
CC	Yes	1.52 (1.13–1.93)	<0.001
CG or GG	Yes	2.40 (1.68–3.63)	<0.001

^a^Adjusted for gender, age, smoke and alcohol consumption status, high fat diet, low fiber diet, TC and HDL

## Discussion

The result of this study indicated that rs1805192- G allele and rs3856806-T allele of PPARG is significantly associated with higher T2DM risk, however, we did not find any association between the others SNPs and T2DM. Although many studies have taken a candidate gene approach to investigate the genetic etiology of T2DM, implicating the potential candidate genes in T2DM risk factors, which include genes for rs1805192 and rs3856806 polymorphism of PPARG [16], however the results of association between rs1805192 and T2DM are inconsistent. Wang et al. [17] demonstrated that the presence of the Ala allele may contribute to improved insulin secretory capacity and may confer protection from T2DM and obesity in the Chinese population. Moreover, a meta-analysis confirmed the association between the PPARG2 Pro12 allele and T2DM, and suggested that patients who carry the Pro12 allele have a 1.27-fold higher risk for developing T2DM than Ala12 carriers. This seemingly modest effect translates into a staggering 25% population-attributable risk because of the higher frequency of the Pro12 allele, especially in Japanese and European populations [18]. However, Bener et al. [19] suggested that no significant association between the Pro12Ala polymorphism of the PPARG2 gene and T2DM in Qatari’s population. Al-Safar et al. [20] also suggested that confirmed that Pro12Ala mutation in PPARG2 is not associated with T2DM risk in this population. In a study for South Africa population, Vergotine et al. [21] reported that the Pro12Ala of PPARG2 is significantly associated with insulin resistance and this polymorphism interacts with IRS1 Gly972Arg, to increase the risk of T2DM. Hahn et al. [22] indicated that our data confirm a beneficial effect of the PPARG1 12Ala allele on insulin resistance and glucose metabolism in PCOS women. Furthermore, the Ala allele appears to be associated with less severe hirsutism, independent of hyperandrogenemia. Tellechea et al. [23] conducted a study for males of Argentinian blood donors of self-reported European ancestry and indicated that healthy men, in particular nonsmokers, carrying the Ala12 allele of PPARG rs1801282 polymorphism, have a high risk for metabolic syndrome (MetS) and insulin resistance (IR). Fan et al. [24] indicated that the genotypes with minor allele variants at the rs1805192, rs709158 and rs3856806 loci are associated with increased LDL-C levels, which was a risk factor of T2DM. Gu et al. [25] also suggested that in the codominant and log-additive models, rs1805192 and rs3856806 were all associated with increased dyslipidemia risk. A study conducted by Phani et al. [26] showed that PPARG2 variants are associated with increased T2DM susceptibility when associated with adiposity in Indian population. These data reflect an association of analyzed PPARG gene polymorphisms with values of insulin, HDL, LDL and total cholesterol which indicates an important role of these genes in lipid metabolism and pathogenesis of T2DM and MetS [16].

T2DM is associated with both environmental and genetic factors. Several metabolic abnormalities are implicated in its pathogenesis, such as obesity and other environmental factors. Several studies have confirmed the association between obesity and T2DM. However, till now, no study focused on impact of gene-environment interaction between PPARG and obesity on T2DM risk in Chinese population. This study investigated the impact of additional PPARG gene-obesity interaction on T2DM risk based on a Chinese population by using GMDR model. We found that a significant two-locus model involving rs1805192 and obesity, indicating a potential gene-environment interaction among rs1805192 and obesity. Obese subjects with CG or GG genotype have the highest T2DM risk, compared to subjects with CC genotype and normal BMI.

Several limitations of this study should be considered. Firstly, More SNPs of PPARG gene should been chosen. The limited SNPs were not sufficient to capture most genetic information of PPARG. Secondly, there was a relatively small sample size in the study, other larger sample studies should be conducted in the future. Thirdly, we did not obtain any information on IR level.

## Conclusions

Our results support an important association between rs1805192 and rs3856806 minor allele (G allele) of PPARG and increased T2DM risk, the interaction analysis shown a combined effect of G- obesity interaction between rs1805192 and obesity on increased T2DM risk.
